# Rare ST-Elevation Myocardial Infarction Mimic: Diabetic Ketoacidosis With Severe Hypercalcemia

**DOI:** 10.7759/cureus.9001

**Published:** 2020-07-04

**Authors:** Tamoor Ahmed, Talha Ahmed, Reyaz Haque

**Affiliations:** 1 Internal Medicine, King Edward Medical University, Mayo Hospital, Lahore, PAK; 2 Internal Medicine, University of Maryland Medical Center, Baltimore, USA; 3 Cardiology, University of Maryland, Baltimore, USA

**Keywords:** diabetic ketoacidosis, acute hypercalcemia, st-elevations, insulin injection, hydration

## Abstract

Diabetic ketoacidosis (DKA) is a common complication in patients with type I and ketosis-prone type II diabetes mellitus. A variety of electrolyte derangements are encountered during the presentation and management of DKA. Hypercalcemia has been rarely reported in DKA, particularly when patients develop severe acidosis. However, we describe a patient with DKA and severe hypercalcemia in the absence of severe acidosis. The hypercalcemia quickly corrected back to normal with the treatment of DKA.

## Introduction

One of the most serious complications of diabetes is diabetic ketoacidosis (DKA). It results from a relative or absolute deficiency of insulin hence resulting in excessive production of keto acids from fat metabolism. Patients encounter various electrolyte and metabolic abnormalities including hypokalemia, pseudohyponatremia, and metabolic acidosis during the presentation and management of DKA [1]. Severe hypercalcemia has been rarely reported in DKA patients. The etiology is usually multifactorial including severe acidosis, dehydration, and other underlying factors like malignancy or granulomatous diseases. The most commonly observed electrocardiogram (ECG) change seen in hypercalcemia is a shortened QTc interval due to accelerated repolarization. In rare cases of severe hypercalcemia, ST-elevations with J-point elevations can also be seen on the ECG [2]. Our case entails the clinical course of a patient who presented with DKA with severe hypercalcemia. The acidosis was not severe to explain the hypercalcemia. An extensive workup for hypercalcemia was negative. In less than two days, with the treatment of DKA, the calcium levels quickly corrected back to normal. This temporal association of DKA and hypercalcemia has been rarely reported in the past [3].

## Case presentation

**Figure 1:**
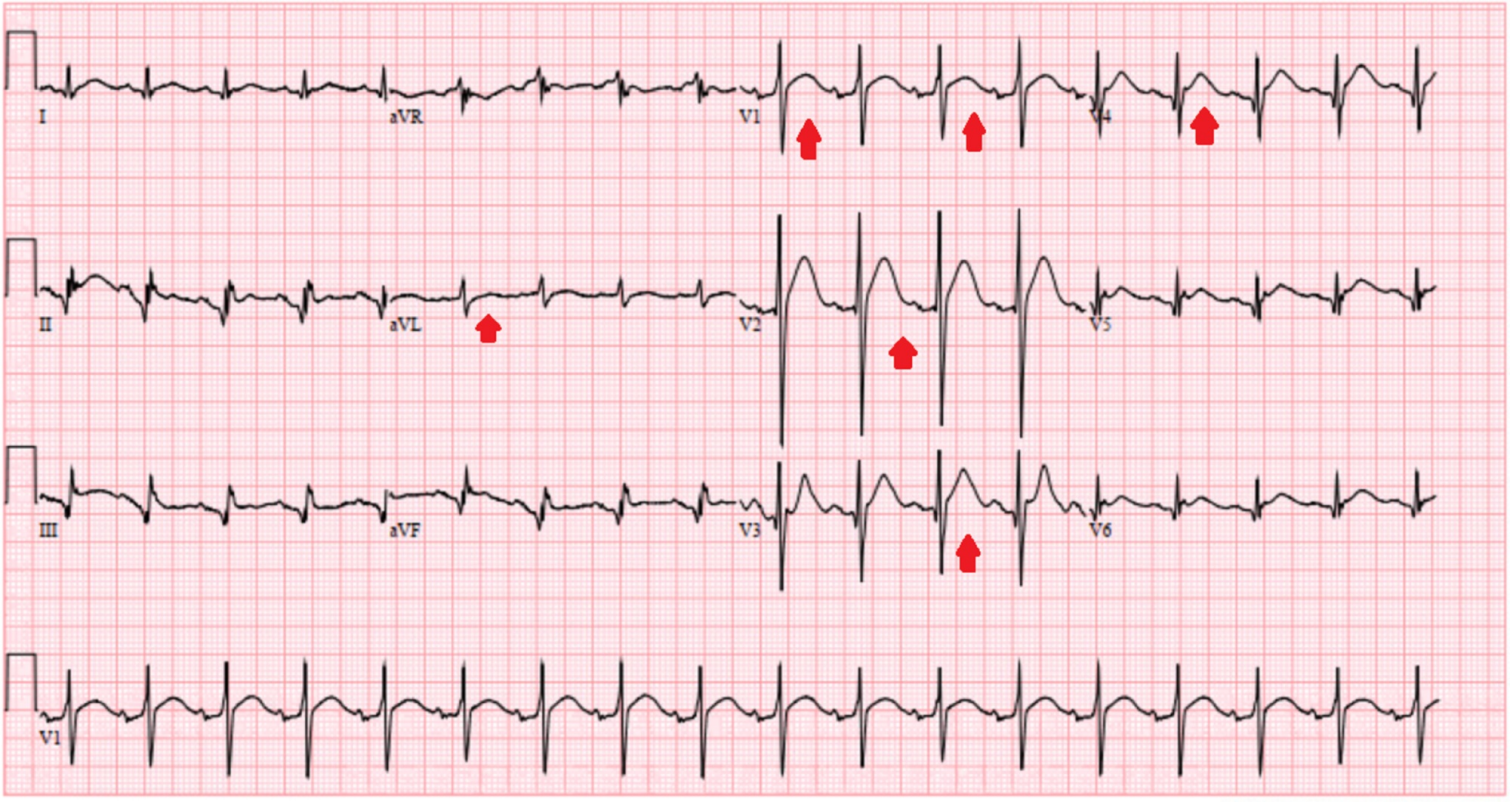
Electrocardiogram showing ST-segment elevation in leads V2-V4 and avL but with no reciprocal changes

A 50-year-old male with history of type I diabetes mellitus presented with nausea, non-bloody emesis, and diffuse abdominal pain. He was not able to take insulin for a week as he ran out of his prescriptions. Vital signs revealed a heart rate of 120 beats per minute, blood pressure of 127/85 mmHg, and the patient was afebrile with normal oxygen saturation (SaO2) of 95% on room air. Abdominal examination revealed generalized tenderness and dry mucous membranes. Initial blood work revealed a white cell count of 24 k/mcL and hemoglobin and hematocrit of 19 g/dl and 53%, respectively, suggesting severe hemoconcentration. Comprehensive metabolic panel showed a blood glucose of 425 mg/dl, the anion gap was elevated at 27 and serum sodium was 127 meq/L with a remarkably elevated calcium of 17.3 mg/dl (normal range 8.5 to 10.5 mg/dl) and serum phosphorous level of 5.9 mmol/L (normal range 2.5 to 4.5 mmol/L). Serum bicarbonate was decreased at 20 meq/L, beta-hydroxybutyrate was elevated at 4.96 mmol/L (normal range of 0.02 to 0.27 mmol/L) and serum potassium level was normal. This combination of laboratory findings suggested a diagnosis of DKA. A thorough infectious workup to evaluate the cause of DKA was unremarkable. An electrocardiogram (ECG) was done which revealed ST-segment elevation in leads V2-V4 and avL with J-point elevations, no reciprocal ST-T wave changes, and normal QTc interval of 440 milliseconds (normal QT interval range 400 to 440 milliseconds) (Figure 1). 

**Figure 2:**
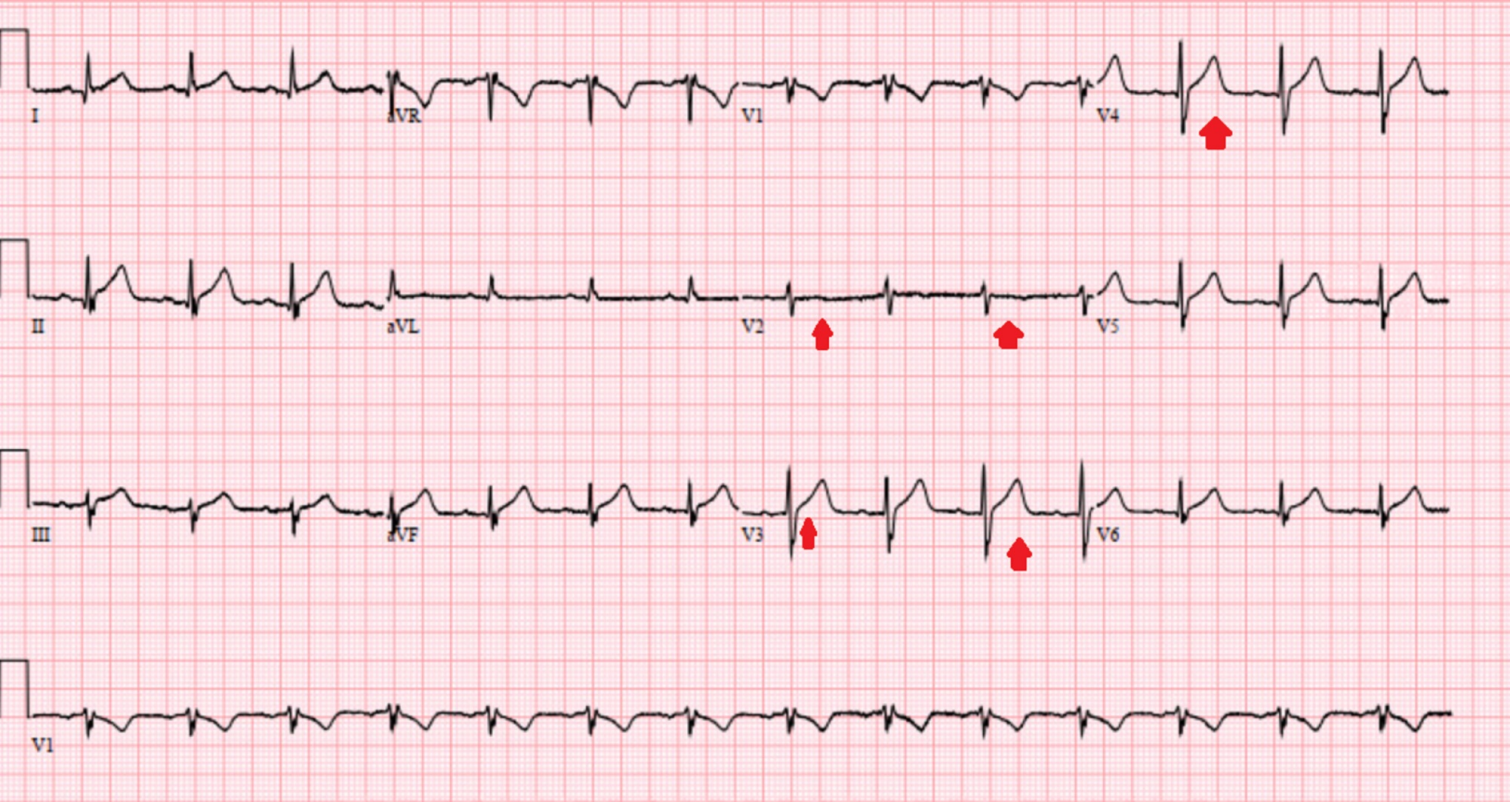
Electrocardiogram showing resolution of the ST changes with the correction of the serum calcium level

These EKG changes were thought to be due to high calcium level as the patient denied any ischemic symptoms and cardiac troponin was minimally elevated at 0.04 ng/ml (reference range of acute myocardial infarction greater than 0.11 ng/ml). Transthoracic echocardiogram suggested a normal cardiac function. The patient was started on intravenous crystalloid boluses of normal saline with a regular insulin drip. Close monitoring of the basal metabolic panel including calcium level was performed. On day two of admission, patients anion gap close to 10 and he was bridged to multiple daily insulin injections. Serum calcium levels trended down to a normal level of 9.5 mg/dl with the resolution of DKA. An extensive workup to evaluate the cause of hypercalcemia was non-revealing. Random urine calcium levels were appropriately elevated at > 17.8 mg/dL while urinalysis showed glucosuria and ketonuria but was otherwise unremarkable. The serum parathyroid hormone was decreased at 5 pg/ml (reference range of 8-54 pg/ml) suggesting a parathyroid independent process, vitamin D, 1,25-dihydroxy was decreased at 17.3 pg/ml (reference range of 19.9-84 pg/ml) and parathyroid hormone-related peptide was normal. In addition, the patient denied taking vitamin D or calcium supplements at home. CT of chest, abdomen, and pelvis was normal excluding malignancy as the cause of hypercalcemia. As a result, the likely cause of this hypercalcemia was attributed to severe DKA and dehydration which corrected with hydration and treatment of DKA. Although the patient was given a dose of zoledronic acid but the calcium corrected before the effects of bisphosphonates could be seen (normally after 48 hours of administration). Due to the rapid correction of serum calcium level back to normal, other medical management (including calcitonin or diuretics) was not warranted. ECG changes also reverted back to normal with the correction of serum calcium levels (Figure 2).

## Discussion

Causes of hypercalcemia can be dissected into either a parathyroid dependent or a parathyroid independent etiology. In the parathyroid independent group, further sub-categories include hypercalcemia of immobilization, malignancy, milk-alkali syndrome, hypervitaminosis D, and granulomatous diseases. Severe hypercalcemia defined as serum calcium levels of more than 14 mg/dl is usually related to malignancy and is refractory to conservative treatment with fluids requiring long-term bisphosphonate therapy [4,5].

DKA is a state of excessive keto acid production that leads to decrease pH of the body. This promotes hypercalcemia by allowing the calcium bound to albumin to set free in the blood as excessive hydrogen ions bind to the albumin instead. This, when compounded by severe dehydration resulting from polyuria caused by osmotic diuresis in diabetics, can theoretically lead to severe hypercalcemia in certain cases [6,7]. This was the likely etiology in our patient considering the extensive negative workup for an alternative cause of high calcium and also with a temporal correlation of resolution of DKA and rapid normalization of serum calcium levels.

The most commonly described ECG change of hypercalcemia is a shortened QTc interval due to rapid repolarization. However, severely elevated calcium levels have been reported to mimic ST-elevation on the ECG [8,9]. Some other ECG changes expected may include abrupt upslope of T wave, PR interval prolongation, and increase the amplitude of the QRS complex. As seen with our patient, these changes revert back to normal with the treatment of hypercalcemia. As opposed to patients with myocardial infarction, these patients don't have reciprocal changes on the ECG [10]. However, a careful history and physical exam should guide us in ruling out the other known causes of ST-elevation on the ECG in these patients [11].

## Conclusions

Hypercalcemia related to DKA is usually a diagnosis of exclusion. Severe acidosis and dehydration can rarely lead to extremely elevated serum calcium levels that rapidly correct to normal with the treatment of DKA. Although the most commonly seen ECG change in hypercalcemia is shortening of QTc interval, ST-elevations without reciprocal changes are expected to be seen in patients with acute severe hypercalcemia. These changes quickly revert back to normal with the treatment and resolution of DKA.
